# Lateral Rectus Myectomy Combined With Medial Rectus Plication for Complete Oculomotor Nerve Palsy: A Case Report With Detailed Postoperative Course

**DOI:** 10.1155/crop/1985508

**Published:** 2026-05-15

**Authors:** Yuna Irie, Kie Iida, Kazusa Kuwano, Masanobu Iida, Takaaki Hayashi, Tadashi Nakano

**Affiliations:** ^1^ Department of Ophthalmology, The Jikei University School of Medicine, Minato-ku, Tokyo, Japan, jikei.ac.jp; ^2^ Department of Ophthalmology, The Jikei University Kashiwa Hospital, Kashiwa, Chiba, Japan, jikei.ac.jp; ^3^ Department of Ophthalmology, Machida Municipal Hospital, Machida-shi, Tokyo, Japan, machida-city-hospital-tokyo.jp/

## Abstract

**Background:**

Lateral rectus myectomy has been reported as an effective and simple surgical option for large‐angle paralytic exotropia. We report a case of complete oculomotor nerve palsy treated with lateral rectus myectomy combined with medial rectus plication, focusing on serial changes in ocular alignment and abduction limitation.

**Case Report:**

A woman in her 20s developed left oculomotor nerve palsy following surgery for an intracranial tumor. At presentation, visual acuity was 20/16 and 20/100, with temporal hemianopia and a central scotoma in the right and left eyes, respectively. She exhibited severe left exotropia exceeding 100 prism diopters (*Δ*), consistent with complete oculomotor nerve palsy, with a marked limitation of adduction graded as −7. Lateral rectus myectomy and 10 mm medial rectus plication were performed on the left eye. The lateral rectus muscle was resected 10 mm posterior to its insertion, and the distal stump was cauterized and allowed to retract into the posterior Tenon′s capsule, intentionally creating a lost muscle. On Postoperative Day 1, ocular alignment improved to 18 *Δ* of esotropia with severe limitation of abduction graded as −4. From 2 months postoperatively, abduction limitation gradually decreased to −2 at 4 months, concomitant with the gradual recurrence of exotropia, after which both findings remained stable. Orbital MRI performed 5 months postoperatively demonstrated posterior reattachment of the lateral rectus muscle via fibrous tissue. At final follow‐up, ocular alignment stabilized at 30 *Δ* of exotropia.

**Conclusions:**

Combined lateral rectus myectomy and medial rectus plication achieved a substantial corrective effect for large‐angle paralytic exotropia due to oculomotor nerve palsy. While this technique represents a safe and effective treatment option, postoperative recurrence may occur due to reattachment of the resected lateral rectus muscle to the globe or orbital pulley, highlighting the need for long‐term follow‐up.

## 1. Introduction

Surgical management of complete oculomotor nerve palsy is among the most challenging forms of paralytic strabismus because only two of the six extraocular muscles, the lateral rectus and superior oblique, remain functional. Consequently, this condition is often refractory, and multiple procedures may be required [1]. Various surgical techniques have been reported [2], including supramaximal lateral rectus recession and medial rectus resection [3, 4], adducting traction sutures [5], orbital wall fixation of the lateral rectus muscle [6], superior oblique transposition [7], nasal transposition of the split lateral rectus muscle [8–10], and ocular fixation to the nasal periosteum [11].

Simpler procedures may be limited by insufficient correction or postoperative recurrence, whereas procedures with greater corrective effect often entail increased invasiveness, technical complexity, and postoperative complications. In 2000, Sato et al. reported lateral rectus myectomy, a simple procedure in which the lateral rectus muscle is resected 10 mm posterior to its insertion to intentionally create a “lost muscle” and markedly reduce the abducting force of the paretic eye [12]. When combined with 10 mm of medial rectus resection, a corrective effect of approximately 55 *Δ* was reported [12].

Despite its simplicity and safety, reports on lateral rectus myectomy remain limited [12, 13], and detailed postoperative courses, particularly serial changes in abduction limitation, have not been well documented. We therefore report a case of large‐angle paralytic exotropia due to acquired complete oculomotor nerve palsy treated with lateral rectus myectomy and medial rectus plication, with emphasis on the postoperative course.

## 2. Case Presentation

A woman in her 20s developed a headache, decreased vision in the left eye, and left oculomotor nerve palsy 2 years earlier. She was diagnosed with an intracranial osteochondroma involving the sellar and suprasellar regions and underwent tumor resection at another institution. Postoperatively, left ptosis and exotropia worsened, and she was referred to our department for surgical correction of strabismus.

**Figure 1:**
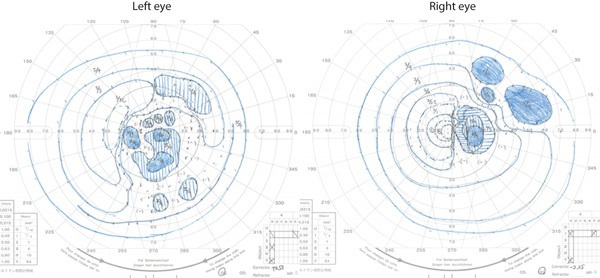
Goldmann kinetic perimetry. A junctional scotoma is shown, with a central scotoma in the left eye and temporal hemianopia in the right eye.

At the initial examination, the best corrected visual acuity was 20/16 in the right eye and 20/100 in the left eye. Goldmann kinetic perimetry revealed a junctional scotoma, characterized by a temporal hemianopia in the right eye and a central scotoma in the left eye (Figure 1).

**Figure 2:**
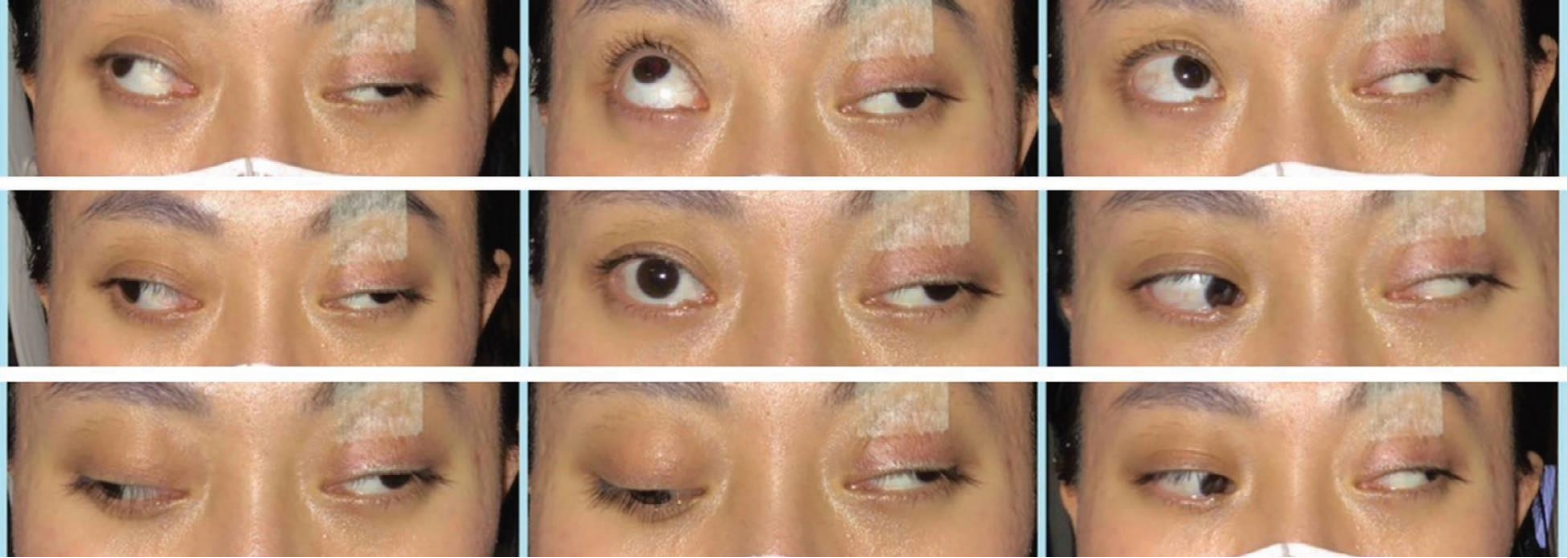
Nine‐gaze photographs before surgery. Preoperative ocular alignment showed exotropia exceeding 100 *Δ* by Krimsky testing. Ocular motility of the left eye demonstrated severe limitation of adduction (−7), supraduction (−4), and infraduction (−4). Left ptosis and pupillary dilation were also present, consistent with complete oculomotor nerve palsy.

The left eye showed ptosis, a dilated pupil, and an absent light reflex. Ocular motility examination demonstrated severe limitation of supraduction (−4), adduction (−7), and infraduction (−4) (graded on a 0 to −8 scale [14]), with exotropia exceeding 100 *Δ* on Krimsky testing, consistent with complete left oculomotor nerve palsy (Figure 2).

**Figure 3:**
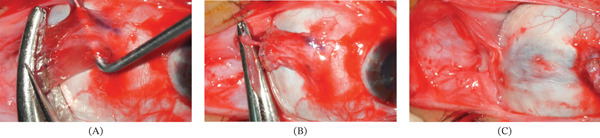
Lateral rectus myectomy in the left eye. (A) The lateral rectus muscle was cauterized and transected 10 mm posterior to its insertion. (B) The distal stump was released without scleral suturing. (C) The distal stump retracted into the posterior Tenon′s space, confirming formation of a lost muscle.

Eighteen months after the initial visit, a 10‐mm lateral rectus myectomy and a 10‐mm medial rectus plication were performed on the left eye. A fornix‐based conjunctival incision was made, and the lateral rectus muscle was exposed. After careful dissection of the surrounding connective tissue, the muscle was clamped 10 mm posterior to its insertion, cauterized, and transected (Figure 3A,B). The distal stump was not sutured to the sclera; instead, it was released and confirmed to have retracted into the posterior Tenon′s space, thereby creating a lost muscle (Figure 3C).

**Figure 4:**
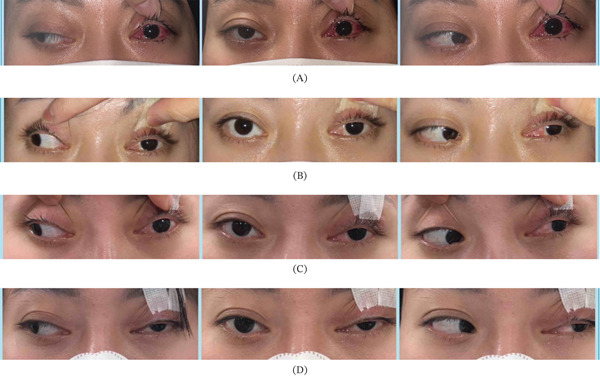
Postoperative course. (A) Postoperative Day 1: Ocular alignment was 18 *Δ* esotropia with marked limitation of abduction (−4). (B) Postoperative Month 2: Ocular alignment shifted to 14 *Δ* exotropia with abduction limitation graded as −3. (C) Postoperative Month 4: Ocular alignment increased to 20 *Δ* exotropia with abduction limitation graded as −2, indicating partial recurrence with lessened abduction limitation. (D) Postoperative Month 8: Ocular alignment remained 20 *Δ* exotropia with stable abduction limitation (−2).

**Figure 5:**
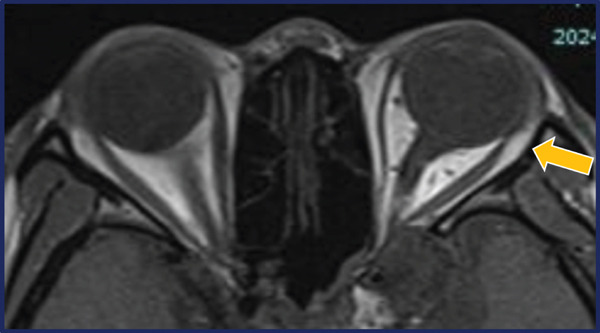
Orbital MRI at 5 months postoperatively. The myectomized left lateral rectus muscle is shown to be reattached to the posterior globe via fibrous tissue. The arrow indicates the site of posterior reattachment.

On Postoperative Day 1, ocular alignment at a distance was 18 *Δ* esotropia, with marked limitation of abduction (−4) (Figure 4A). At 2 months postoperatively, ocular alignment shifted to 14 *Δ* exotropia, with slight recovery of abduction (−3) (Figure 4B). At 4 months, abduction limitation decreased to −2, and exotropia increased to 20 *Δ*, indicating partial recurrence (Figure 4C). Orbital MRI performed 5 months postoperatively demonstrated reattachment of the myectomized lateral rectus muscle to the posterior globe via fibrous tissue (Figure 5). At 8 months postoperatively, both abduction limitation and ocular alignment remained stable (Figure 4D). At the final follow‐up, 16 months after surgery, ocular alignment was 30 *Δ* exotropia.

## 3. Discussion

In this case, combined 10‐mm lateral rectus myectomy and 10‐mm medial rectus plication improved a preoperative exotropia of more than 100 *Δ* to approximately 30 *Δ*. Given the simplicity of the technique and its suitability for local anesthesia, this combined procedure represents an effective option for managing large‐angle paralytic exotropia due to complete oculomotor nerve palsy with minimal invasiveness.

We focused particularly on postoperative changes in abduction limitation. Immediately after surgery, the operated eye showed severe abduction limitation (−4), consistent with intraoperative confirmation that the lateral rectus muscle had retracted into the posterior Tenon′s space as a lost muscle. However, abduction began to recover at 2 months postoperatively and partially recovered by 4 months, accompanied by the recurrence of exotropia. By 8 months, both abduction limitation and ocular alignment had stabilized, with no substantial change through the final follow‐up. Overall, the corrective effect in this case was at least 70 *Δ*.

In the literature, Farvardin et al. reported outcomes in 13 patients with complete oculomotor nerve palsy and exotropia ranging from 50 to 120 *Δ* who underwent lateral rectus myectomy and maximal medial rectus resection, with additional superior oblique transposition in nine cases [13]. At 6 months postoperatively, 11 of 13 patients achieved alignment within 10 *Δ* of orthotropia [13]. In the four cases without superior oblique transposition, the corrective effect ranged from 45 to 52 *Δ* [13]. Sato et al. reported a corrective effect of approximately 55 *Δ* [12]. Together, these findings suggest that lateral rectus myectomy can provide a substantial corrective effect by intentionally creating a lost muscle and thereby markedly weakening the abducting force of the paretic eye.

Nevertheless, in the present case, postoperative orbital MRI demonstrated posterior reattachment of the myectomized lateral rectus muscle to the posterior globe via fibrous tissue, consistent with findings reported in previous studies [12, 13]. This reattachment likely resulted in partial functional recovery of the lateral rectus and contributed to recurrent exotropia during follow‐up. Postoperative diplopia did not occur in this patient, likely because of reduced preoperative visual acuity and marked visual field defects in the affected eye. In patients in whom diplopia is not a concern and cosmetic alignment in primary gaze is the main goal, performing a more posterior myectomy may help limit reattachment to the globe or orbital pulley and reduce postoperative recurrence.

An additional advantage of lateral rectus myectomy is that it preserves future surgical options. Although multiple surgeries are often required in cases of complete oculomotor nerve palsy, secondary procedures such as superior oblique transposition can still be performed if undercorrection occurs. In addition, medial rectus plication rather than resection was chosen in this case to preserve anterior segment circulation and reduce the risk of anterior segment ischemia. However, in patients with good visual acuity, inducing abduction limitation and potentially limiting adduction may expand the range of postoperative diplopia; therefore, careful patient selection is essential. Furthermore, while lateral rectus myectomy effectively produces profound abduction limitation, its quantitative predictability may be limited. Nevertheless, surgical outcomes may be influenced by factors such as the duration of palsy and the severity of lateral rectus contracture. In cases with long‐standing palsy and marked contracture, stronger posterior retraction of the resected muscle may limit postoperative reattachment and reduce the likelihood of recurrent exotropia. In addition, severe lateral rectus contracture may make other procedures, such as split lateral rectus transposition, technically difficult [15], making lateral rectus myectomy a potentially useful alternative. However, no previous reports, including the present case, have specifically described its use in patients with severe lateral rectus contracture. Further studies are needed to clarify its indications and postoperative behavior.

## 4. Conclusions

Combined lateral rectus myectomy and medial rectus plication is a simple and effective surgical approach to minimize the abducting force of the paretic eye by intentionally creating a lost lateral rectus muscle, thereby providing substantial correction for large‐angle exotropia associated with oculomotor nerve palsy. However, postoperative recurrence may occur due to posterior reattachment of the myectomized lateral rectus muscle, necessitating long‐term follow‐up and consideration of additional surgical intervention.

## Author Contributions

All the authors meet the International Committee of Medical Journal Editors (ICMJE) criteria and have significantly contributed to this manuscript. Y.I., K.I., K.K., and M.I. are responsible for the design, concept, intellectual content, manuscript preparation, literature search, and data acquisition/analysis. K.I., T.H., and T.N. are responsible for manuscript proofreading, editing, review, and supervision.

## Funding

No funding was received for this manuscript.

## Ethics Statement

For this type of study, ethical approval is not required. The article is written according to the World Medical Association Declaration of Helsinki.

## Consent

Informed consent for publication of this case report was obtained. No personal identifying information is disclosed in this report.

## Conflicts of Interest

The authors declare no conflicts of interest.

## Data Availability

The data that support the findings of this study are available from the corresponding author upon reasonable request.
